# Method for detecting and quantitating capture of organic molecules in hypervelocity impacts

**DOI:** 10.1016/j.mex.2021.101239

**Published:** 2021-01-23

**Authors:** Bahar Kazemi, James S. New, Matin Golozar, Laura D. Casto, Anna L. Butterworth, Richard A. Mathies

**Affiliations:** a Department of Chemistry, University of California, Berkeley, United States; b School of Physical Sciences, University of Kent, United Kingdom; c Space Sciences Laboratory, University of California, Berkeley, United States

**Keywords:** Biosignature detection, Enceladus plume capture methods, Quantitative epifluorescence microscopy, Enceladus organic analyzer

## Abstract

Enceladus is a prime candidate in the solar system for in-depth astrobiological studies searching for habitability and life because it has a liquid water ocean with significant organic content and ongoing cryovolcanic activity. The presence of ice plumes that jet up through fissures in the ice crust covering the sub-surface ocean, enables remote sampling and in-situ analysis via a fly-by mission. However, capture and transport of organic materials to organic analyzers presents distinctive challenges as it is unknown whether, and to what extent, organic molecules imbedded in ice particles can be captured and survive hypervelocity impacts. This manuscript provides a fluorescence microscopic method to parametrically determine the amount of an organic fluorescent tracer dye, Pacific Blue™ (PB) deposited on a metallic surface. This method can be used to measure the capture and survival outcomes of terrestrial hypervelocity impact experiments where an ice projectile labeled with Pacific Blue impacts a soft metal surface. This work is an important step in the advancement of instruments like the Enceladus Organic Analyzer for detecting biosignatures in an Enceladus plume fly-by mission.

An apparatus consisting of a substrate humidification shroud coupled with an epifluorescence microscope with CCD detector is developed to measure the intensity of quantitatively deposited Pacific Blue droplets under controlled humidity.

Calibration curves are produced that relate the integrated fluorescence intensity of humidified PB droplets on metal foils to the number of PB molecules deposited.

To demonstrate the utility of this method, our calibrations are used to analyze and quantitate organic capture and survival (up to 11% capture efficiency) following ice particle impacts at a velocity of 1.7 km/s on an aluminum substrate.

## Graphical abstract

Logarithmic calibration curve relating the integrated fluorescence intensity of humidified PB droplets to the number of PB molecules. Each humidified droplet is shown with a different color and placed at its corresponding concentration and intensity. These data were obtained using the 10x objective.

**Figure:**
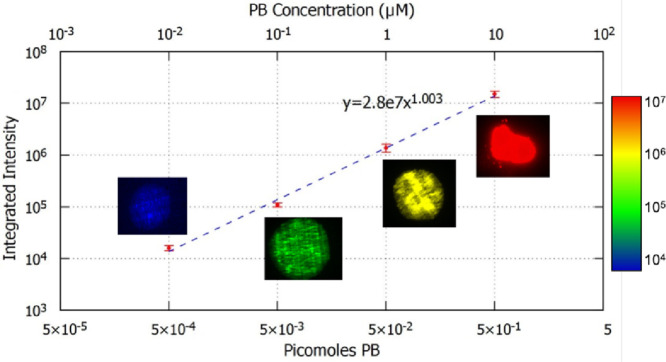

Specification table

**Table:** 

Subject Area	Chemistry
More specific subject area	*Astrobiology*
Method name	*Calibration of a Method for Quantitating Capture of Organic Molecules in Hypervelocity Impacts*
Name and reference of original method	*If applicable, include full bibliographic details of the main reference(s) describing the original method from which the new method was derived.*
Resource availability	https://imagej.nih.gov/ij/ https://www.thermofisher.com/order/catalog/product/P10163#/P10163

## Method details

### Background

Enceladus, an icy moon of Saturn, harbors a global liquid water ocean [1,2] with salinity similar to Earth's oceans [3], that resides above a rocky core [4] and beneath an icy crust. Plumes of water vapor and ice grains venting through four prominent warm troughs in the south polar terrain (SPT) ice sheet [5,6] erupt thousands of kilometers into space [7] and are believed to be driven by ongoing hydrothermal activity at the ocean-core interface [8], [9], [10]. The analysis of Cassini data suggests the presence of simple organic substances in plume material [11], [12], [13], and more recent studies indicate the observation of complex macromolecular organic compounds in the emitted ice grains [14,15]. In view of the presence of a liquid water ocean with significant organic content and ongoing cryovolcanic activity, Enceladus is a prime candidate in the solar system for in-depth astrobiological studies searching for habitability and for life. This new information suggests that a fly-by mission through the plume that gathers organic molecule-containing ice for organic analysis for biosignatures would be scientifically very interesting and likely productive [16]. However, to accomplish this goal, milligrams of plume particle material must be captured to enable ppb detection by typical organic analyzers [17]. Also, for many formats, the encounter will be in the “hypervelocity” regime, characterized with high shock pressures and temperatures. Thus it is important to understand the possible impact degradation and/or alteration of the desired organic molecules [18].

There is significant literature on organic compound capture and survival after hypervelocity impact. The Stardust mission collected thousands of particles by direct impact into low-density silica aerogel during fly-by through the tail of Comet Wild-2 [19,20]. Fujishima et al. performed terrestrial hypervelocity impacts on ultra-low-density silica aerogel using peptide-rich particles to assess the capture and survival of short peptides post impact [21]. While trace organic molecules and cometary amino acids [22], [23], [24] were detected, aerogels are extremely hard to work with for trace organic analysis [25], [26], [27]. Parnell et al. demonstrated the survival of organic biomarkers in projectile fragments and ejecta after hypervelocity impact using siltstone and steel projectiles and siltstone, sand, and water as the impact target [28]. Burchell et al. observed the transfer and survival of organic content in frozen projectiles to a variety of target types after hypervelocity impacts [29]; however, they did not determine the efficiency of organic transfer or the degree of degradation in the transfer process. Burchell and Harriss examined the possibility of distinguishing aromatics from aliphatics during high-speed impacts [30]. More recently, New et al. [31] used polymethyl methacrylate (PMMA) microspheres as ice-simulants to study their capture and the behavior of organic bonds post-impact on several metal targets. They demonstrated that organics remain chemically unmodified and survive impacts of up to several kilometers per second. The logical next step is to quantitatively determine the efficiency of organic capture for ice particle impacts and to measure the extent of possible degradation.

The purpose of this study is to develop a method that enables the quantitative determination of the capture efficiency of organic molecules entrained in ice projectiles following a hypervelocity impact with a metal surface. This method serves as the calibration for light gas gun experiments, in which frozen projectiles doped with an organic fluorescent tracer dye, Pacific Blue™, were shot onto a variety of metal surfaces [32]. For the calibration portion of this project, aluminum foil was selected as the mock capture surface as it is a soft inert metal that allows capture of particles and can be easily cleaned to avoid organic contamination [31]. A quantitative fluorescence microscopic method was developed to analyze films of intact PB molecules deposited on an aluminum surface and calibration plots were generated relating the integrated fluorescence signal intensity to the number of PB molecules. Since the fluorescence intensity is sensitive to molecular degradation of the quantum yield and fluorescence spectrum, it is a good measure of the extent of captured and unaltered model organic material.

The method and calibrations were then successfully used to analyze and quantitate organic capture and survival following ice particle impacts at velocities of 1, 2, and 3 km/s [32]. This result is an important step in establishing the feasibility of using the Enceladus Organic Analyzer and other chemical analysis instruments to sensitively probe the Enceladus plume for biosignatures [16,17].

## Materials and method

The major steps in the method include preparing PB solutions at various concentrations and depositing PB droplets of known small volume onto an aluminum substrate. After dehydrating the droplets, the sample was inserted into the humidification apparatus equipped with an epifluorescence microscope, an oblique white light illumination system and a CCD camera. The sample was then humidified to a humidity determined to make the PB strongly fluorescent. Then the integrated fluorescence intensity of the spot was measured to determine the fluorescence intensity per Pacific Blue molecule on the surface.

### Experimental apparatus

**Fig. 1:**
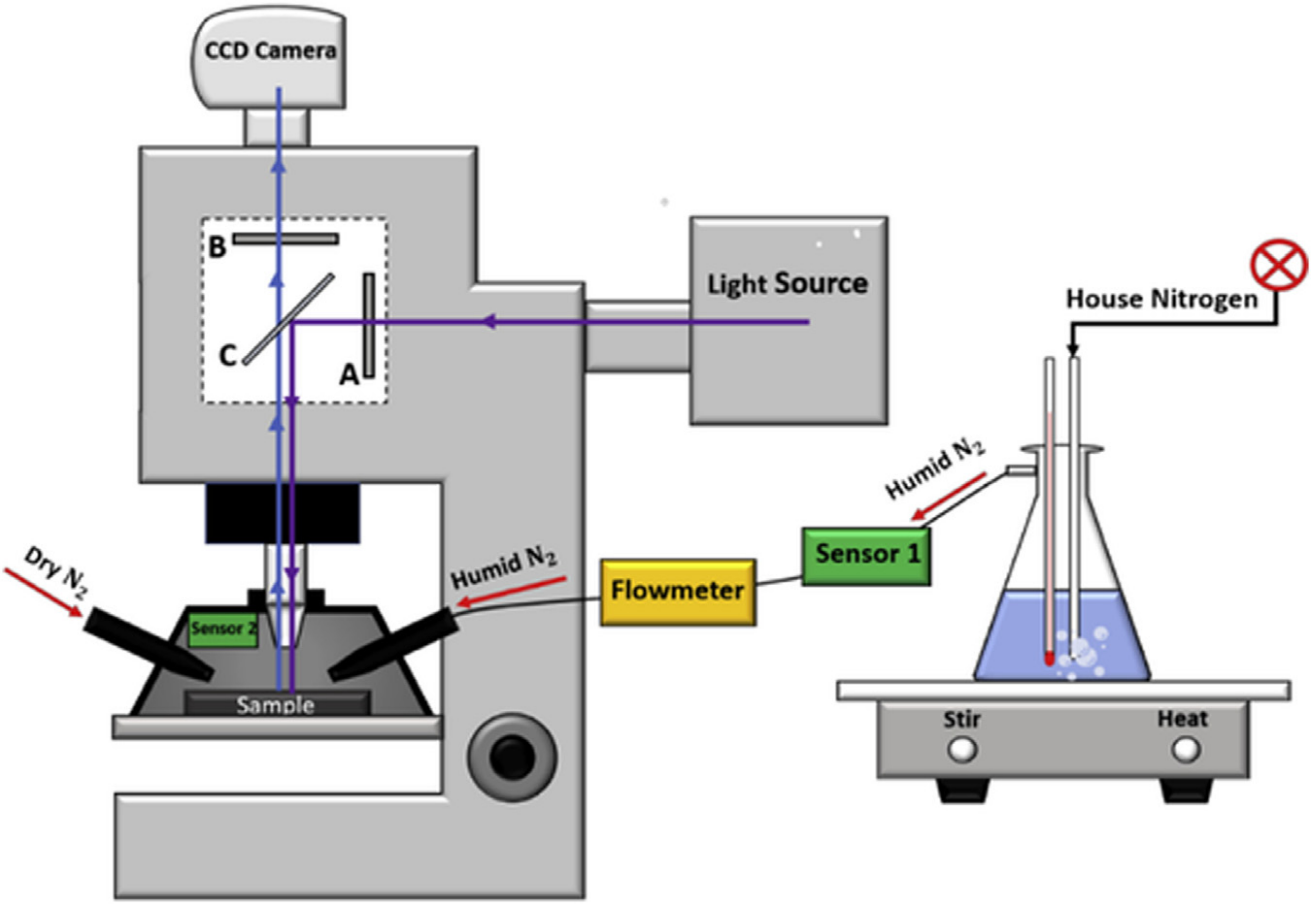
Schematic of the epifluorescence microscope and hypervelocity target humidification apparatus. Dry nitrogen bubbles through the water and exits as humidified nitrogen. The sample is enclosed in a 3-D printed black shroud that ensures the sample experiences uniform humidity. The filter cube includes a 405-nm band-pass excitation filter (A), a 435-nm long-pass emission filter (B) and the dichroic mirror (C) appropriate for Pacific Blue emission.

Fig. 1 presents a schematic of the epifluorescence microscope and hypervelocity target and calibration standard humidification apparatus. A side-arm flask filled half-way with water and heated to 38 ± 1°C served as a gas humidification bath. Dry nitrogen was bubbled through the water with a tubular glass frit and humidified nitrogen exited through the side-arm of the flask and tubing to the sample which was enclosed in a 3-D printed black shroud that surrounds the sample and the objective. The black shroud reduced ambient light interference with the fluorescence measurement. The shroud also ensured that the sample experienced a uniform humidity which was monitored by two humidity sensors (one placed at the side-arm exit and one placed inside the chamber). The gas flow rate through the TSI digital flowmeter (1400 series) and the water temperature were adjusted to achieve a humidity of 70% measured inside the shroud. The measurement cycle consisted of first flowing dry nitrogen through a second inlet into the shroud to dehumidify the sample for bright-field and background fluorescence image acquisition. Then the humidified nitrogen flow is turned on to humidify the substrate to the desired level for PB fluorescence measurements.

A Nikon Eclipse E800 epifluorescence microscope equipped with a 100-watt mercury lamp, a 5 Megapixel Media Cybernetics Evolution MP color CCD camera, an S Plan Fluor ELWD 20X/0.45 and a Plan 10x/0.25 Nikon objective were used for image acquisition. The microscope filter cube included a 405-nm band-pass excitation filter and a 435-nm long-pass emission filter optimal for Pacific Blue emission intensity. Additionally, a separate oblique white light illumination system, not shown in the figure, was used to obtain bright light images of the sample.

### Sample preparation

Pacific Blue™ succinimidyl ester (PB), purchased from ThermoFisher Scientific, was dissolved in DMF (dimethylformamide) to produce a 10 mM stock solution. Serial dilutions of the stock solution in 30 mM sodium tetraborate, pH 9.3 were performed to produce 10 µM, 1 µM, 100 nM, and 10 nM PB solutions. A 2-inch circle of cardboard covered with rubber silicone tape was used to create a soft substrate to support the aluminum foil for droplet deposition. Using a soft support substrate ensures that the tip of the syringe contacts and stays in contact with the surface and facilitates a complete transfer of droplet from the tip of the syringe onto the surface. Aluminum foil was placed shiny side down on the rubber tape and cleaned by gently rubbing with lens tissue wetted with acetone. This process also smoothed the surface of the foil. If the foil is cleaned too aggressively the increased surface hydrophobicity prevents the droplets from wetting the surface, whereas a somewhat hydrophilic surface enables reproducible droplet deposition with a uniform size when dehydrated.

**Fig. 2:**
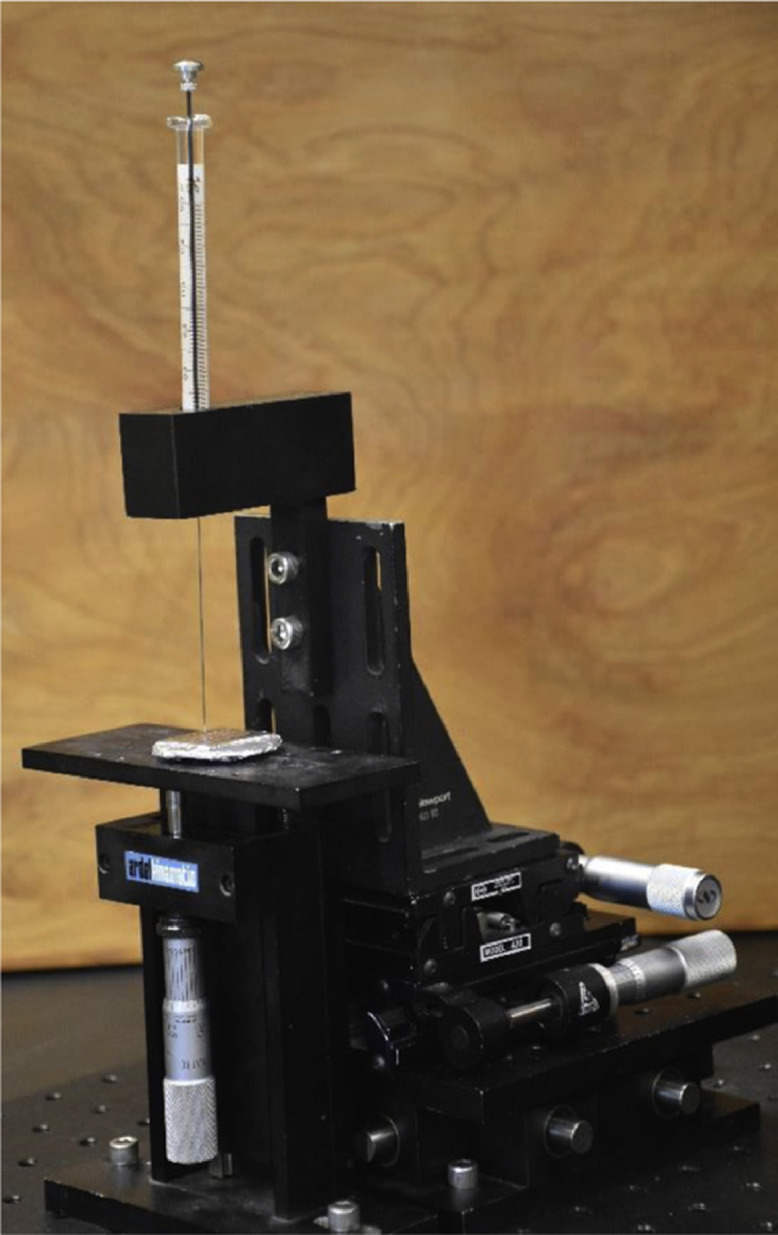
Custom micro-droplet spotter made to deposit standard droplets on aluminum foil substrate.

To perform an accurate and precise droplet deposition, a custom micro-droplet spotter was assembled using three orthogonal linear stages as shown in Fig. 2. A 3-D printed syringe holder was mounted to hold the syringe perpendicular to the sample surface. This setup allows accurate positioning of the syringe relative to the foil surface with a nominal accuracy of 32 µm in the vertical direction. To avoid cross-contamination and false-positive fluorescent signals, four 0.5 µL Hamilton syringes (7000 Series, point style 3) were used, one for each PB concentration. A fifth syringe was used to deposit blank control droplets that only contained 30 mM sodium tetraborate buffer. To generate the calibration curve for the 10x objective, 50 nL droplets (three replicates at each concentration as well as the blank control) were deposited onto the surface forming droplets of ~500 µm diameter. Since the 10x objective had insufficient magnification to detect craters smaller than 20 µm in diameter, the same calibration procedure was repeated using the 20x objective that enabled detection of craters down to 5 µm in size. To generate the calibration curve for the 20x objective, which has a smaller field of view, 5 nL droplets were deposited onto the surface producing droplets of ~250 µm in diameter. For these smaller droplets, the number of replicates at each concentration was increased to 24 to reduce the error due to different tip-to-substrate volumetric transfer efficiency and droplet size. After preparation, the 10x and 20x standard arrays were stored in a dark room at ambient temperature for 24 h to dry the PB droplets. This process was performed to mimic the dehydration that occurs during and after hypervelocity ice particle impacts and to ensure that the measurements are performed on uniformly rehydrated substrates.

### Image acquisition and analysis

**Fig. 3:**
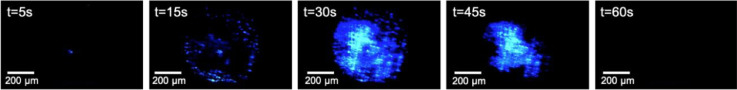
The fluorescence intensity of PB depends strongly on the humidity. Selected frames from a video of a 50 nL 100 µM PB droplet using 10x objective. As the humid nitrogen is introduced through the shroud (3.5 ± 0.1 L/min) the PB molecules start glowing. The signal intensity continues increasing and intense fluorescing emission is observed when the humidity reaches the optimal value of 70% at *t* = 30 s with no evident condensation. When dry nitrogen is introduced, the signal rapidly drops and is unobservable at *t* = 60 s.

A set of experiments were performed to determine the humidity at which PB droplets emit optimal fluorescence signals. The fluorescence intensity of a PB film is a strong function of humidity as demonstrated by the enclosed video and the five fluorescence images presented in Fig. 3. Dry PB films are only weakly fluorescent but can be reversibly hydrated to produce intense fluorescence. In the actual ice shot experiments, this dependence was found to be very useful in determining which residues are true PB depositions as opposed to stray background fluorescence.

To determine the optimum PB hydration point, three 1 µM PB droplets were deposited on an aluminum substrate and stored in a dark room for 24 h to evaporate the solvents. The substrate was placed under the shroud and the humidity of the foil was reduced to 10% (the starting point) by flowing dry nitrogen through the shroud. A flow of humid nitrogen gas at 3.5 ± 0.1 L/min was then introduced to increase the humidity. The water temperature was kept at 38 ± 1 °C throughout the experiment and the exposure time was set to 1 s. Images were acquired with every 20% increase in the humidity up to 90%. PB emission increased until the humidity in the chamber reached 70%; further increase in humidity did not cause an increase in the fluorescence but did result in undesired condensation on the aluminum foil. Therefore, 70% humidity was selected as the optimal value for image acquisition for calibration and for impact target analysis. The fluorescence intensity of PB also depends on the pH of the solution; it is a maximum at basic pH 9 due to the presence of electron-withdrawing fluorine atoms in its molecular structure [33]. Therefore, all subsequent experiments were performed with pH 9.3 sodium tetraborate buffer, a pH at which photodestruction was also found to be minimized.

**Fig. 4:**
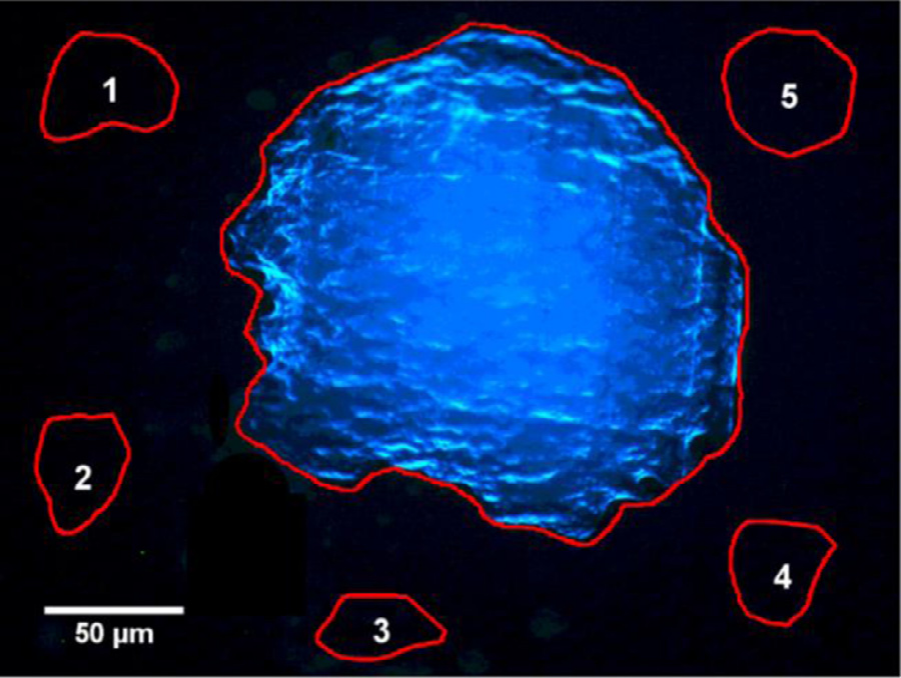
A humidified 1 µM PB droplet image acquired using an S Plan Fluor ELWD 20X/0.45 Nikon objective with an exposure time of 1 s. This image was stacked to RGB and its contrast was enhanced allowing 0.1% of the pixels to become saturated. The red outlines define the ROI of the humidified droplet as well as five PB-free regions in the vicinity of the humidified droplet (numbered 1–5) used to measure the signal intensity and background emission, respectively.

Calibration plots were generated for both the 10x and 20x microscope objectives. Following rehumidification a fluorescence image of each droplet was acquired using MediaCybernetics Image-Pro-Express software. The exposure time was set to 10 s for 10 nM and 100 nM PB droplets. To avoid pixel saturation at higher concentrations, the exposure time was reduced to 1 s and 0.1 s for droplets at 1 µM and 10 µM, respectively. Each fluorescence image was imported to ImageJ using Bio-Formats Importer plugin (https://imagej.net/Bio-Formats#Bio Formats_Importer) and was stacked and split into its RGB components. The analysis was performed on the blue channel which solely contains the PB emission data. The first step was to define Regions of Interest (ROI) by outlining the humidified droplet as illustrated in Fig. 4. The ROI was used to determine the area (A) and the signal mean intensity (${\bar{I}}_{s}$). The background emission (${\bar{I}}_{\bar{B}}$) was obtained by averaging the mean intensity of five PB-free ROIs in the vicinity of the humidified droplet and this average background was subtracted from the droplet's mean signal intensity. The resulting corrected signal intensity was multiplied by the area of the ROI and normalized with respect to exposure time. Eq. (1) presents how the integrated intensity was calculated for each droplet.

(1) $$I=\;\frac{\left({\bar{I}}_{s}-{\bar{I}}_{\bar{B}}\right)A}{s}$$

where ${\bar{I}}_{s}$ is the signal mean intensity, ${\bar{I}}_{\bar{B}}$ is the averaged background mean intensity, A is the area of the droplet in $\mu m^{2}$, and s is the exposure time in seconds. The number of PB molecules in each droplet was calculated by knowing the concentration and the deposited volume and was converted to picomoles using:

(2) $$\text{Picomoles}\;\text{of}\;\text{PB}=C\;\times \;V\;\times 10^{-3}$$

where C is the PB concentration in μM, and V is the deposited volume in nL. Tables 1 and 2 (*supplementary materials*) provide the measurements and analysis of the standard droplets using the 10x and 20x objectives, respectively.

### 10x calibration curve

**Fig. 5:**
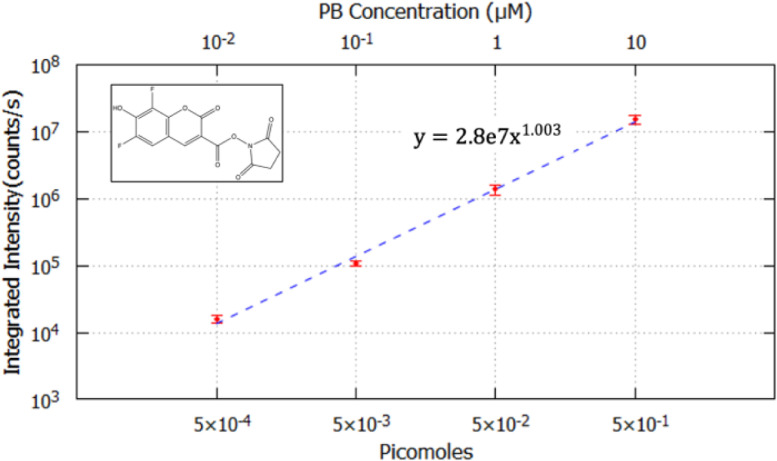
Integrated intensity plotted against picomoles PB on a logarithmic scale using the 10X objective. The secondary x-axis represents PB concentration in 50 nL droplets. Three replicate data sets were obtained at each concentration using the 10x objective following drying and rehydration at 70% humidity. A power function ($y=ax^{b}$) was fit to the data for quantitation with following parameters: a=2.8e7±8.6e4 (0.28% error), b=1.003±0.004 (0.36% error). The molecular structure of Pacific Blue succinimidyl ester is shown on the top left.

The 10x aluminum substrate contained 3 data replicates at each concentration. The integrated intensity was calculated for each droplet, averaged at each concentration and plotted against picomoles of PB as shown in Fig. 5. The secondary x-axis represents the equivalent PB concentration of each picomolar value. The error bars represent the standard deviation (1σ, *n* = 3) of Integrated Intensity. A linear correlation between the integrated intensity and picomoles of PB was observed as expected. This relationship could be used to determine the number of PB molecules in a droplet region at any given intensity spanning this observation range.

### 20x calibration curve

**Fig. 6:**
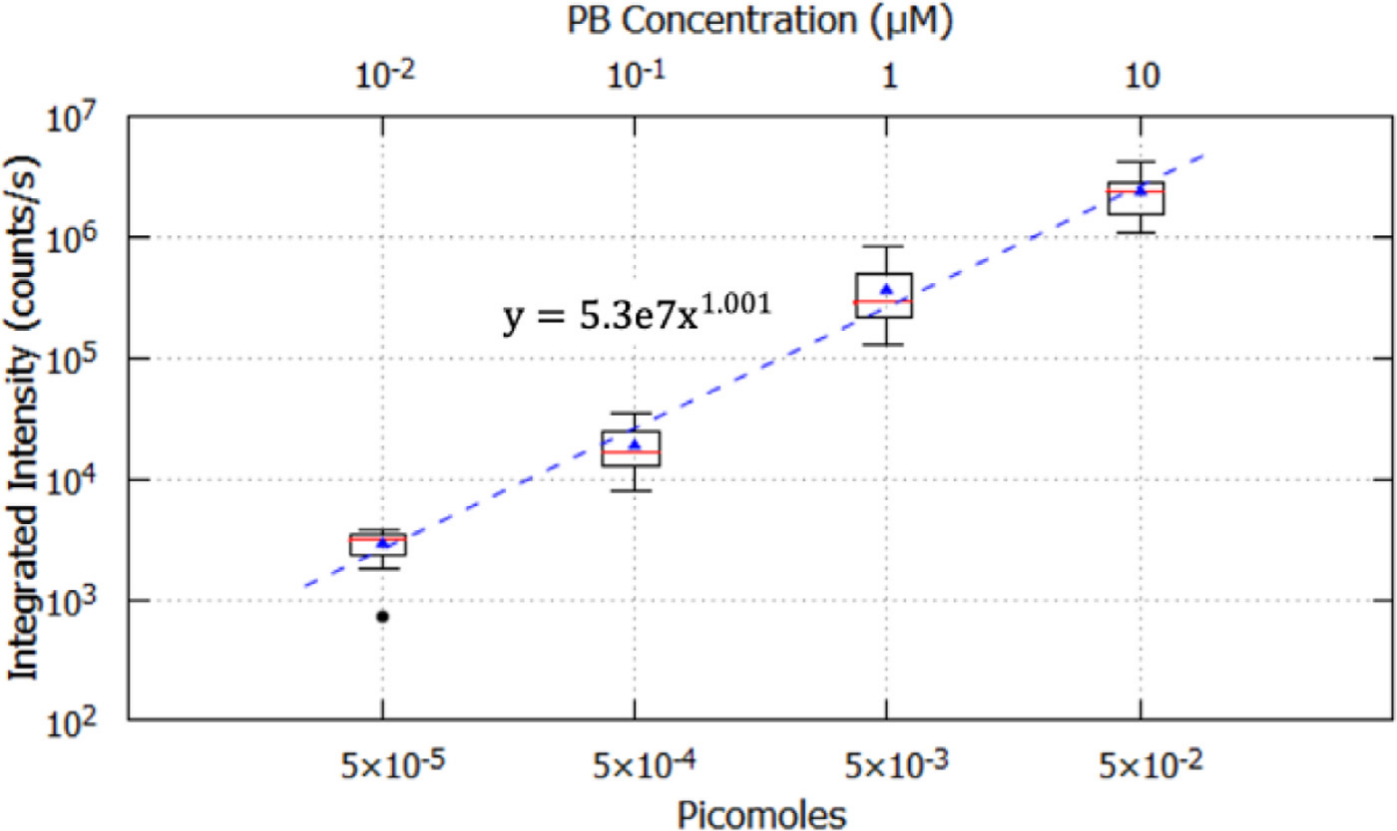
Boxplots showing integrated intensity plotted against picomoles PB on a logarithmic scale for the 20x calibration. The secondary x-axis represents PB concentration in 5 nL droplets. Replicate data sets (24) were obtained at each concentration using the 20x objective following drying and rehydration at 70% and an average of 15 droplets were analyzed at each concentration. The solid red lines represent the median, the blue triangles represent the mean, and the solid black points represent the outliers. A power function ($y=ax^{b}$) was fit to the data for quantitation with following parameters: a=5.3e7±2.6e6 (9.7% error), b=1.001±0.032 (4% error).

The 20x aluminum substrate contained 24 data replicates at each concentration. Only the droplets that fit within field of view of the CCD camera (an average of 15 droplets at each concentration) were analyzed to develop the 20x calibration plot (Fig. 6). For each droplet, integrated intensity was calculated and plotted against picomoles of PB using boxplots on a logarithmic scale. The solid red lines represent the median and the blue triangles represent the mean. Whiskers extend from the ends of the box to the most extreme data point that is no more than 1.5 IQR (Turkey method). Outliers that are more than 1.5 IQR are represented with black filled circles. The median and mean values of integrated intensity are very similar at each PB concentration with the smallest difference of 2% at 10 nM and the largest difference of 28% at 1 µM. A linear correlation between the mean values of integrated intensity and picomoles PB was observed indicating that the signal intensity increases linearly with PB concentration. This relationship can be interpolated to obtain the number of PB molecules at any given integrated intensity.

### Capture efficiency example

The two-stage light gas gun (LGG) at the University of Kent [34,35] was used to perform a series of hypervelocity impact experiments to evaluate the utility and practicality of this method. The facility was modified to fire frozen projectiles containing 100 µM PB suspended in 30 mM sodium tetraborate buffer solution. Impact craters ranging from several µm to mm in diameter were formed on the capture surface. A more detailed account of the complete set of impact experiments and procedures will be found in New et al. [32]

**Fig. 7:**
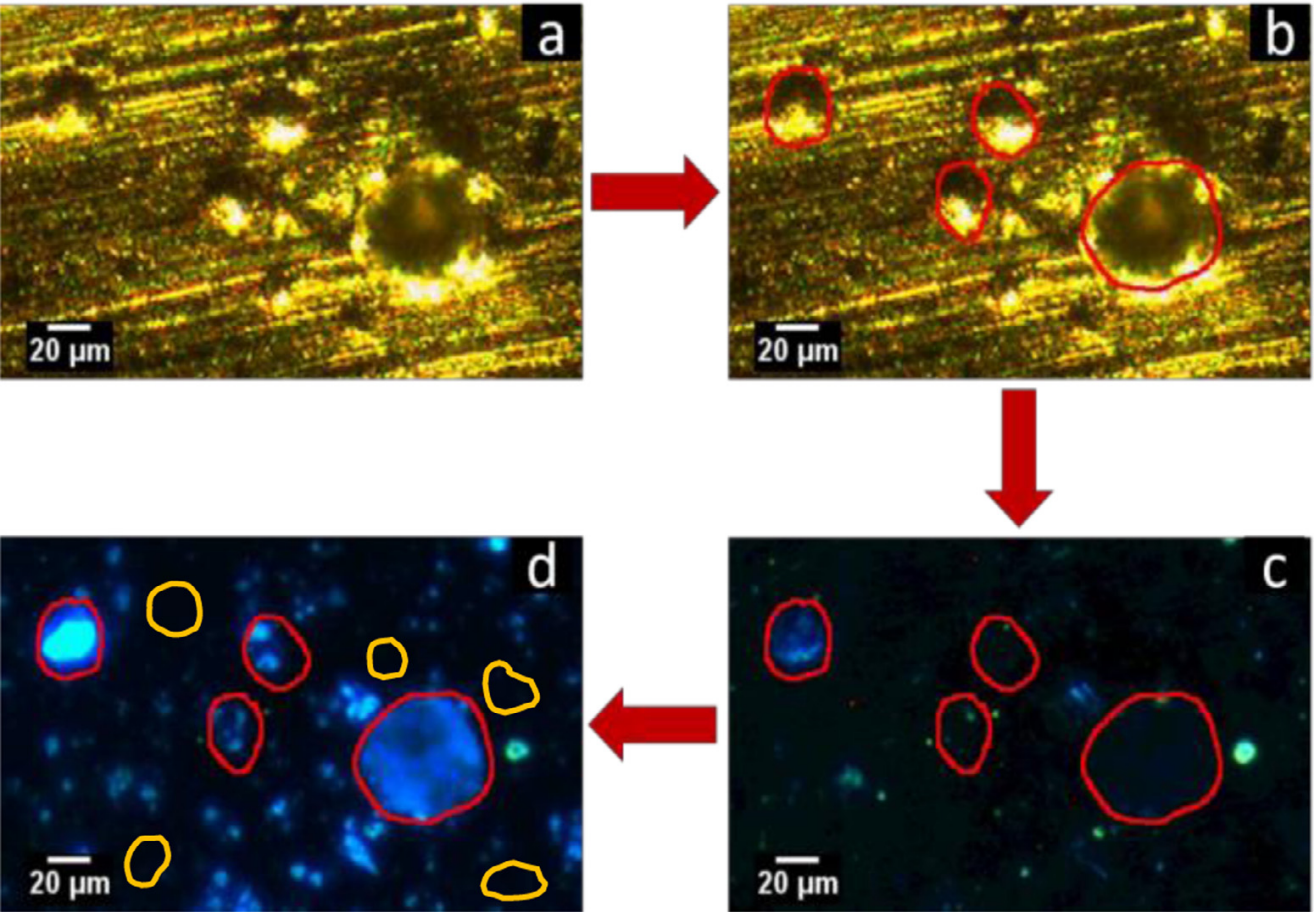
Crater analysis on aluminum foil formed at 1.7 km/s impact velocity using the 10x objective. (a,b) Bright-field image showing craters and regions of interest (ROI) outlined with red. (c) Low humidity fluorescence image with superimposed ROIs showing artifacts and weak dry PB emission in craters. (d) Humidified (70%) fluorescence image with superimposed red ROIs showing the appearance of intense PB emission inside the craters and yellow ROIs showing the PB-free regions used to measure background emisison. Contrast was enhanced in image c and d, allowing 0.1% of the pixels to become saturated.

To analyze each impact crater, three images were acquired. A bright-field and an epifluorescence image were acquired after pumping dry nitrogen over the foil to reduce the humidity to below 10%. Then, humid nitrogen was pumped through the shroud and an epifluorescence image was acquired at 70% humidity. Both epifluorescence images were acquired at 10 s exposure time, and the bright-field image was acquired at 1 s exposure time. The bright-field image was used to locate the craters, identify the ROIs and measure major and minor axes of the craters using the fit-ellipse tool in ImageJ as seen in Fig. 7a and 7b. The original ROIs were superimposed on the low humidity fluorescence image to identify any fluorescence artifact or residue inside the crater that might significantly affect the measured signal intensity. The humidified fluorescence image represented in Fig. 7d demonstrated a striking increase in PB emission specifically in the craters. The fully hydrated fluorescence image was split into its RGB components and the mean intensity of the craters as well as three PB-free regions in the vicinity of each crater was measured on the blue channel which solely contains the PB emission data. Finally, the integrated intensity was obtained using Eq. (1) and the number of PB molecules in each crater was calculated using calibration equations from the plots shown in Figs. 5 and 6, depending on the objective magnification used for image acquisition.

Organic capture and survival efficiency is defined as the percentage of PB molecules in the impacting particle that were captured and successfully detected in a crater. To calculate the capture efficiency, it is necessary to determine the number of PB molecules in the impacting particle. A detailed study has been done by New et al. [31] that provides a means of calculating the impactor diameter based on the crater size. They used PMMA microspheres with well-defined diameters to establish particle-crater diameter calibrations at different impact velocities for several capture surface materials including aluminum. Since the mechanical properties of PMMA are similar to ice, these calibrations were used in our study to estimate the ice-particle diameter based on the crater dimensions. The diameter of each crater was calculated by averaging major/minor axes obtained from the fit-ellipse tool in ImageJ and the diameter of the impacting ice-particle was obtained using particle-crater calibrations. Assuming that the ice-particles were spherical, the volume of the impacting particle was calculated and multiplied by its original concentration in ice projectiles (100 µM) to find out the number of PB molecules in the impactor. Eq. (3) shows how the number of picomoles PB molecules in the impactor was calculated based on its original concentration in the ice projectile:

(3) $$\text{Picomoles}\;\text{of}\;\text{PB}\;\text{in}\;\text{the}\;\text{Impactor}=\frac{1}{6}\pi D^{3}\times 10^{-7}$$

where D is the impacting ice-particle diameter in µm. Capture efficiency is then determined by:

(4) $$\text{Capture}\;\text{Efficiency}\;\left(\%\right)=\frac{\text{Picomoles}\;\text{of}\;\text{PB}\;\text{in}\;\text{the}\;\text{crater}}{\text{Picomoles}\;\text{of}\;\text{PB}\;\text{in}\;\text{the}\;\text{impactor}}\times \;100$$

## Results

**Fig. 8:**
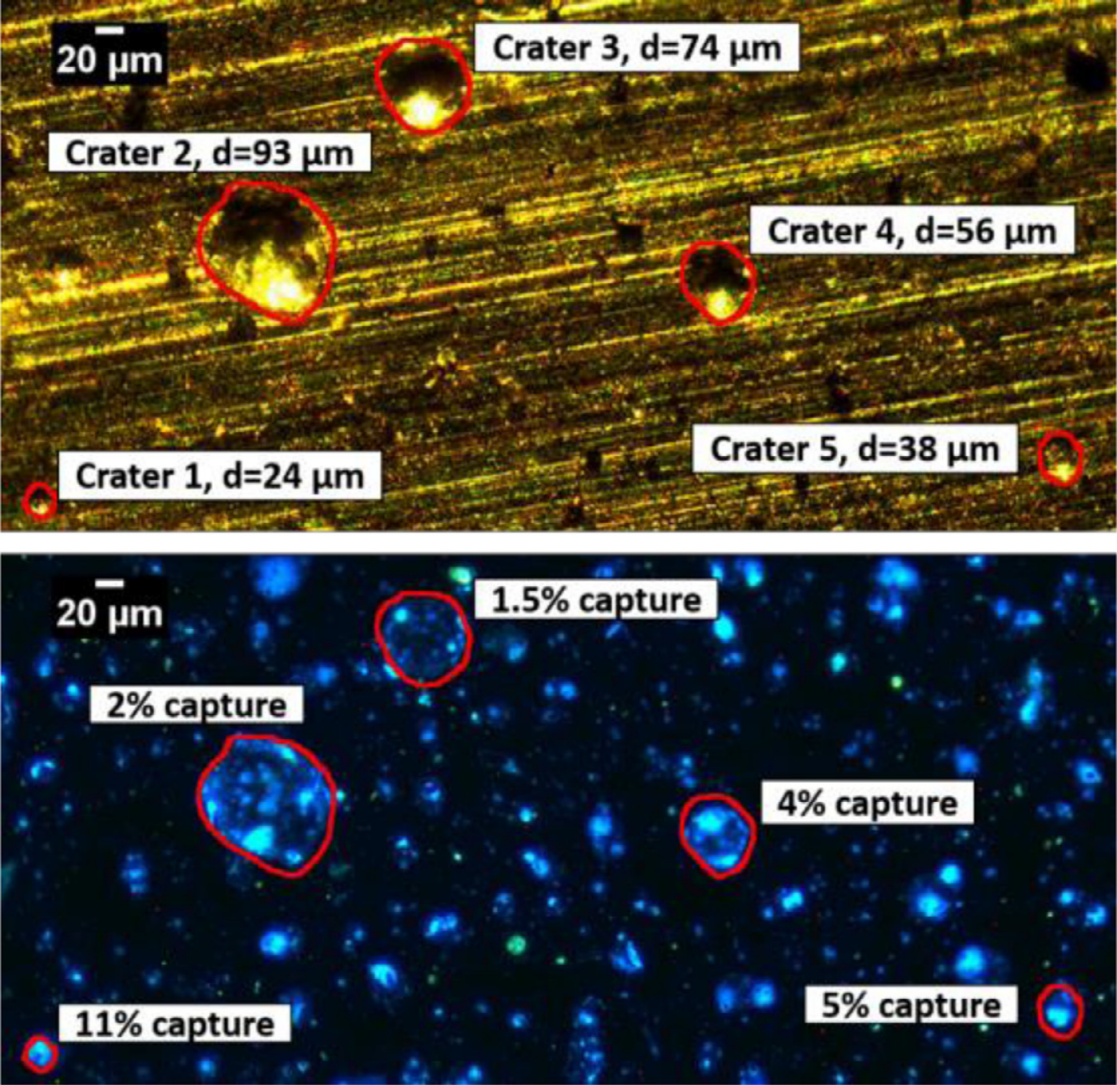
Bright-field (top) and humidified fluorescence (bottom) images of craters on aluminum foil formed during a 1.7 km/s impact velocity. Images were acquired using a 10x objective. Intense fluorescing PB emission is observed inside the outlined craters labeled with particle diameters and capture efficiencies. Contrast was enhanced in the bottom image, allowing 0.1% of the pixels to become saturated.

**Fig. 9:**
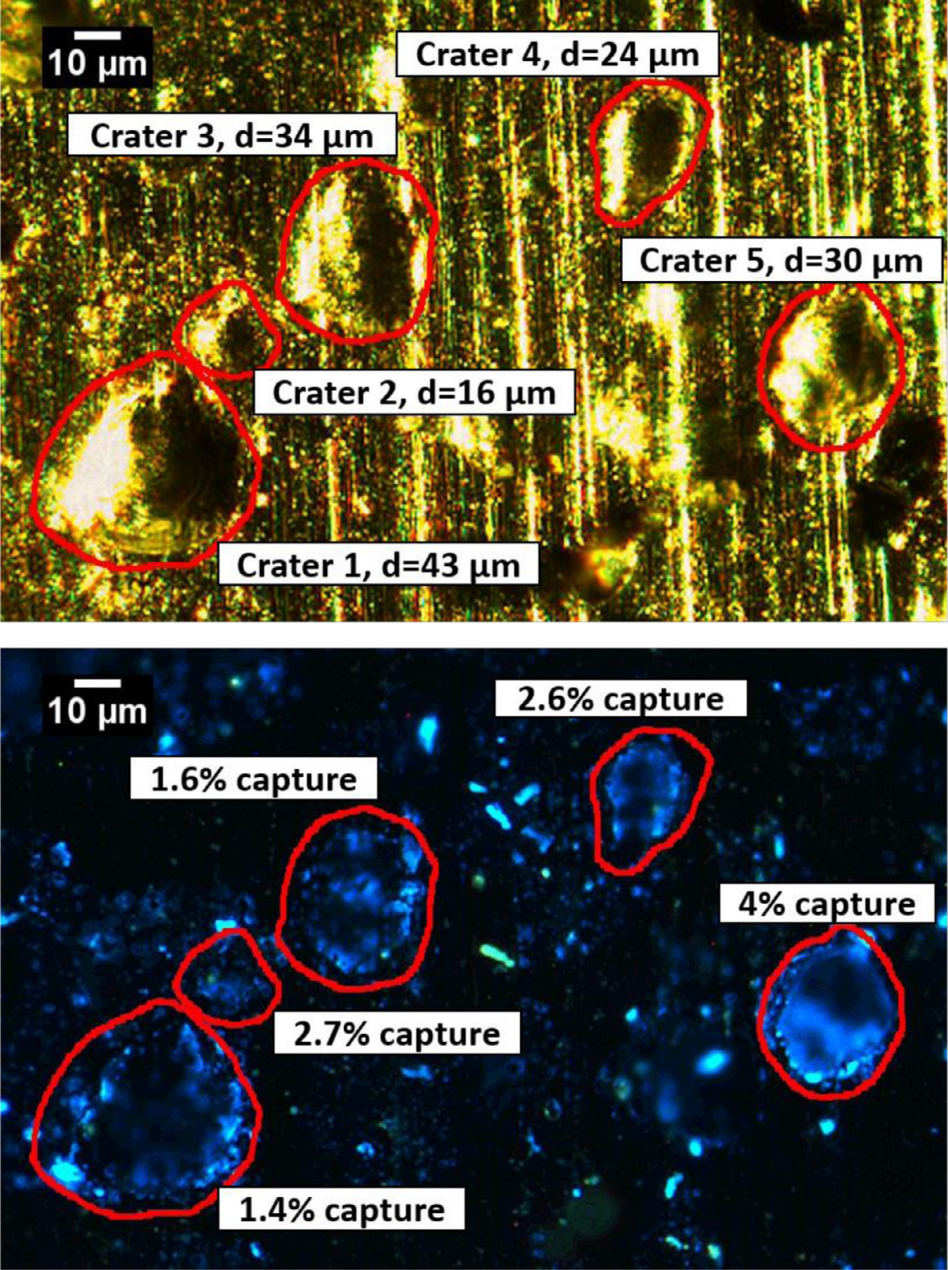
Bright-field (top) and humidified fluorescence (bottom) images of several craters on aluminum foil formed during a 1.7 km/s impact velocity. Images were acquired using a 20x objective enabling examination of smaller craters. Intense PB emission is observed inside the outlined craters labeled with particle diameters and capture efficiencies. Contrast was enhanced in the bottom image, allowing 0.1% of the pixels to become saturated.

To illustrate the utility of our method, crater analysis was carried out on aluminum for an impact velocity of 1.7 km/s using 10x and 20x objectives. Figs. 8 and 9 illustrate several representative craters ranging from 16 to 93 µm on aluminum substrate. Once humidified, intact PB molecules are readily detected inside the craters, indicating successful capture and impact survival, and capture efficiency can be quantitated based on the fluorescence intensity and the particle diameter obtained from the bright-field image. In this set of experiments at 1.7 km/s, the capture efficiencies ranged from 1 to 11%. The method described here has been used to perform a very detailed experimental study of the relationship between capture efficiency, impact velocity, and particle diameter; this work is reported separately [32].

## Conclusion

Sampling pristine ocean constituents during Enceladus plume fly-by transects involves hypervelocity impacts, which involve high shock pressures and temperatures that may lead to degradation and/or alteration of the captured organic molecules. Although several studies demonstrated organic molecule survival during hypervelocity impacts, the efficiency of organic capture was qualitative and the degree of degradation undetermined. We developed a novel quantitative fluorescence microscopic method to determine the capture efficiency of organic molecules entrained in ice projectiles following hypervelocity impact. The utility of this method is demonstrated here through the quantitative analysis of organic capture and survival following representative ice particle impacts at 1.7 km/s on aluminum substrates. The 10x and 20x calibration curves were generated to detect and quantitate organic capture in craters ranging from 5 µm to 500 µm in diameter. While the 10x objective is unable to detect craters smaller than 20 µm, the 20x objective and calibration enable detection and analysis of craters down to 5 µm in size. Such small craters are of particular interest as the majority of Enceladus plume mass at an elevation of 50 km consists of particles with diameters <5 µm that, based on particle-crater diameter calibrations at various velocities for aluminum [31], are expected to form craters of 5 µm and above in a fly-by mission [36].

Our method and the results have important implications for the design, optimization and selection of future Enceladus missions. This method and the more detailed publication that follows [32] provide detailed experimental evidence supporting the idea originally presented by Mathies et al. [16] that unmodified organic molecules from the Enceladus plume can be efficiently collected by impacts into soft metal surfaces for chemical analysis. Combining this collector with instrumentation to dissolve and transport the captured analytes to a high sensitivity organic analyzer like the Enceladus Organic Analyzer [16] will produce a powerful approach for biosignature detection at Enceladus and other icy moons.

## Declaration of Competing Interest

The authors declare that they have no known competing financial interests or personal relationships that could have appeared to influence the work reported in this paper.

## Appendix Supplementary materials

Image, application 1

Image, video 2
