# Supplementary material for: Method for detecting and quantitating capture of organic molecules in hypervelocity impacts
Source: MethodsX. 2021 Jan 23;8:101239. doi: 10.1016/j.mex.2021.101239 (PMC8374173; doi:10.1016/j.mex.2021.101239)
Supplement: Supplementary file 1 [file mmc1.docx]

**Method for Detecting and Quantitating Capture of Organic Molecules in Hypervelocity Impacts**

*Bahar Kazemi, James S. New, Matin Golozar, Laura D. Casto, Anna L. Butterworth, Richard A. Mathies*

A series of high-velocity and hypervelocity impact

experiments were carried out us ing the light gas gun

(LGG) at the University of Kent (described by Burchell

et al. 1999; Hibbert et al. 2017), as seen and illustrated

in Fig. 1. This particular LGG facility was selected due

to its ability to accelerate a wide range of particles, both

size and composition, up to velocities of ~7.5 km s

1

and offer ﬂexibility in target conﬁguration and

temperature

A series of high-velocity and hypervelocity impact

experiments were carried out us ing the light gas gun

(LGG) at the University of Kent (described by Burchell

et al. 1999; Hibbert et al. 2017), as seen and illustrated

in Fig. 1. This particular LGG facility was selected due

to its ability to accelerate a wide range of particles, both

size and composition, up to velocities of ~7.5 km s

1

and offer ﬂexibility in target conﬁguration and

temperature

A series of high-velocity and hypervelocity impact

experiments were carried out us ing the light gas gun

(LGG) at the University of Kent (described by Burchell

et al. 1999; Hibbert et al. 2017), as seen and illustrated

in Fig. 1. This particular LGG facility was selected due

to its ability to accelerate a wide range of particles, both

size and composition, up to velocities of ~7.5 km s

1

and offer ﬂexibility in target conﬁguration and

temperature

**Supplementary Materials**

This appendix contains a description of supplementary methods and experiments used to investigate the effect of several parameters on the fluorescence of PB.

**The effect of repeated drying and rehydration on PB fluorescence.** The effect of dehydration on PB fluorescence was assessed during image acquisition for several craters. Certain craters were captured in more than one image. These craters were in close proximity to other craters of interest and remained in the field of view of microscope after slightly moving the sample to capture the adjacent crater image, and thus experienced dehydration and rehydration more than once. For such craters, our data indicates that the intensity changes only within an average of 1.5% after repeated drying and rehydration. Therefore, drying and rehydrating does not degrade or affect PB intensity measurements.

**The effect of nitrogen flowrate and humidity on PB fluorescence.** At flowrates higher than 3.5 ± 0.1 L/min, the humidity inside the shroud increases at a faster rate resulting in surface condensation and not enough time for image acquisition at a known humidity. It was found that 3.5 ± 0.1 L/min was the optimal flowrate at which the humidity inside the shroud increases at a rate that provides enough time for image acquisition and good control of the humidity level. This flowrate was then selected to determine the optimum PB hydration point. Three 1 µM PB droplets were deposited on an aluminum substrate and stored in a dark room for 24 hours to evaporate the solvents. The substrate was placed under the shroud and the humidity of the foil was reduced to 10% (the starting point) by flowing dry nitrogen through the shroud. A flow of humid nitrogen gas at 3.5 ± 0.1 L/min was then introduced to increase the humidity. The water temperature was kept at 38 ± 1 ˚C throughout the experiment and the exposure time was set to 1 second. Images were acquired with every 20% increase in the humidity up to 90%. PB emission increased until the humidity in the chamber reached 70%; further increase in humidity did not cause an increase in the fluorescence but did result in undesired condensation on the aluminum foil. Therefore, 70% humidity was selected as the optimal value for image acquisition for calibration and for impact target analysis.

**The effect of condensation on PB fluorescence.** Condensation involves formation of large water droplets on the surface. Since there is no control of the volume or location of these water droplets, they distort the deposited droplets and would destroy the information in the foils used for the impact experiments. When humidity was increased above 70%, it did not cause an increase in the fluorescence but did result in undesirable condensation on the aluminum foil. Therefore, 70% humidity was selected as the optimal value to avoid undesirable condensation on the foil.

**Fluorescence of wet vs. dry PB droplets.** To compare the fluorescence of a PB droplet before it dries with the intensity of a PB droplet at 70% humidity, an experiment was performed to acquire and compare images of several droplets before they dry and when they are rehydrated at 70% humidity. Since the size and volume of standard droplets for 10x (50 nL and 500 $\mu$m) and 20x (5 nL and 250 $\mu$m) objectives were very small they dried shortly after deposition onto the surface and it was not possible to measure the fluorescence of the sample before it dried. Therefore, this experiment was done using a 4x objective which had a wider field of view and enabled observation of larger PB droplets (millimeter range) which did not dry immediately after deposition. Our results indicate a 10% decrease in fluorescence signal of rehydrated droplets compared to the wet droplets. Since our calibration experiments and shot experiments are both performed on rehydrated samples, this deviation does not affect the accuracy of our calibrations.

**The effect of photo-destruction on PB fluorescence.** Photo-destruction typically occurs when fluorescent molecules transition from an excited singlet state to the excited triplet state and are temporarily or permanently quenched. To determine the effect of photo-destruction, three 1 µM PB droplets were deposited on an aluminum substrate and prepared for testing as described in the humidity experiment above. The humidity was kept at 70% and the droplets were exposed to UV light throughout the experiment. The experiment duration was 12 minutes. Images were acquired every 30 seconds at an exposure time of 10 seconds. Our results indicate that after 2 minutes of nonstop exposure to UV, the intensity of rehydrated droplets reduces by only 10%, while after 12 minutes, the intensity reduces by about 50%. Therefore, at an exposure time of 10 seconds, which is the maximum exposure time in our experiments, photo-destruction is not significant.

**Calibration curves.** 10x and 20x calibration curves are shown in Figure 5 and Figure 6 of the paper’s main body, respectively. The difference in the calibration coefficients is due to the difference in numerical aperture (NA) and objective magnification (M). Image fluorescence brightness is proportional to ${NA}^{4}/M^{2}$, and it also depends on the light transmission through the optical components. Based on the information provided on Nikon global website (<https://www.microscope.healthcare.nikon.com/products/optics/cfi-plan-achromat-series>), for the 10x and 20x objectives used in this experiment the transmission at 435nm is 0.8 and 0.9, respectively. Therefore, the brightness for the 20x objective is about 2.1 times greater than the 10x objective ($\frac{(\frac{{0.45}^{4}}{{20}^{2}})}{(\frac{{0.25}^{4}}{{10}^{2}})}\approx2.1$). Similarly, the ratio of calibration coefficients for 20x and 10x objective is equal to 1.9 which is only about 10% lower than what we expected. The camera settings are similar for both objectives. Thus, the two calibration curves are consistent with expectation.

**Supplementary Tables**

| **Table 1**. Table of data for standard droplets at different PB concentrations using a 10x objective. |  |  |  |  |  |  |  |
| --- | --- | --- | --- | --- | --- | --- | --- |

| Concentration | Deposited Volume  (nL) | Picomoles PB | Exposure Time  (s) | Area  $\boldsymbol{(\mu m)}^{\mathbf{2}}$ | Mean Intensity | Mean Background | (Integrated Intensity)/s |
| --- | --- | --- | --- | --- | --- | --- | --- |
| 10 nM | 50 | 0.0005 | 10 | 424,104 | 1.499 | 1.075 | 17,971 |
| 10 nM | 50 | 0.0005 | 10 | 409,664 | 1.409 | 1.052 | 14,625 |
| 10 nM | 50 | 0.0005 | 10 | 398,175 | 1.422 | 1.042 | 15,121 |
| 100 nM | 50 | 0.005 | 10 | 355,432 | 4.673 | 1.900 | 98,553 |
| 100 nM | 50 | 0.005 | 10 | 413,385 | 4.681 | 1.986 | 111,397 |
| 100 nM | 50 | 0.005 | 10 | 408,055 | 4.722 | 1.869 | 116,428 |
| 1 $\boldsymbol{\mu}$M | 50 | 0.05 | 1 | 404,563 | 4.924 | 0.916 | 1,621,591 |
| 1 $\boldsymbol{\mu}$M | 50 | 0.05 | 1 | 420,561 | 3.703 | 0.993 | 1,139,824 |
| 1 $\boldsymbol{\mu}$M | 50 | 0.05 | 1 | 376,882 | 4.398 | 0.780 | 1,363,747 |
| 10 $\boldsymbol{\mu}$M | 50 | 0.5 | 0.1 | 438,204 | 3.964 | 0.914 | 13,366,311 |
| 10 $\boldsymbol{\mu}$M | 50 | 0.5 | 0.1 | 432,486 | 4.996 | 0.933 | 17,572,976 |
| 10 $\boldsymbol{\mu}$M | 50 | 0.5 | 0.1 | 426,350 | 4.298 | 0.935 | 14,339,230 |

**Table 2.** Table of data for standard droplets at different PB concentrations using a 20x objective.

| Concentration  (μM) | Deposited Volume  (nL) | Picomoles PB | Exposure Time  (s) | Area  $\boldsymbol{(\mu m)}^{\mathbf{2}}$ | Mean Intensity | Mean Background | (Integrated Intensity)/s |
| --- | --- | --- | --- | --- | --- | --- | --- |
| 10 nM | 15 | 0.00005 | 10 | 33,188 | 1.926 | 0.94 | 3,272 |
| 10 nM | 15 | 0.00005 | 10 | 14,713 | 1.077 | 0.575 | 739 |
| 10 nM | 15 | 0.00005 | 10 | 45,394 | 1.263 | 0.5158 | 3,392 |
| 10 nM | 15 | 0.00005 | 10 | 27,070 | 1.618 | 0.9506 | 1,807 |
| 10 nM | 15 | 0.00005 | 10 | 39,087 | 1.389 | 0.6146 | 3,027 |
| 10 nM | 15 | 0.00005 | 10 | 19,720 | 2.537 | 0.9148 | 3,199 |
| 10 nM | 15 | 0.00005 | 10 | 43,123 | 1.194 | 0.485 | 3,057 |
| 10 nM | 15 | 0.00005 | 10 | 34,248 | 1.806 | 0.9466 | 2,943 |
| 10 nM | 15 | 0.00005 | 10 | 30,962 | 1.686 | 0.9284 | 2,346 |
| 10 nM | 15 | 0.00005 | 10 | 39,201 | 1.883 | 0.9562 | 3,633 |
| 10 nM | 15 | 0.00005 | 10 | 43,248 | 1.388 | 0.638 | 3,244 |
| 10 nM | 15 | 0.00005 | 10 | 44,446 | 1.828 | 0.9676 | 3,824 |
| 10 nM | 15 | 0.00005 | 10 | 49,975 | 1.585 | 0.8982 | 3,432 |
| 10 nM | 15 | 0.00005 | 10 | 39,231 | 1.202 | 0.65 | 2,166 |
| 100 nM | 15 | 0.0005 | 10 | 37,780 | 10.099 | 0.953 | 34,554 |
| 100 nM | 15 | 0.0005 | 10 | 46,816 | 5.623 | 0.7978 | 22,590 |
| 100 nM | 15 | 0.0005 | 10 | 13,809 | 6.476 | 0.8588 | 7,757 |
| 100 nM | 15 | 0.0005 | 10 | 26,715 | 14.148 | 0.9818 | 35,174 |
| 100 nM | 15 | 0.0005 | 10 | 23,394 | 6.189 | 0.7872 | 12,637 |
| 100 nM | 15 | 0.0005 | 10 | 29,343 | 6.573 | 1.0106 | 16,322 |
| 100 nM | 15 | 0.0005 | 10 | 25,145 | 5.905 | 0.9134 | 12,551 |
| 100 nM | 15 | 0.0005 | 10 | 26,644 | 6.332 | 0.8902 | 14,499 |
| 100 nM | 15 | 0.0005 | 10 | 31,213 | 4.507 | 0.8228 | 11,499 |
| 100 nM | 15 | 0.0005 | 10 | 38,727 | 8.717 | 0.9146 | 30,217 |
| 100 nM | 15 | 0.0005 | 10 | 19,888 | 9.458 | 0.9882 | 16,844 |
| 100 nM | 15 | 0.0005 | 10 | 51,717 | 4.338 | 0.8648 | 17,962 |
| 100 nM | 15 | 0.0005 | 10 | 35,270 | 5.339 | 0.9058 | 15,636 |
| 100 nM | 15 | 0.0005 | 10 | 29,864 | 9.201 | 0.966 | 24,593 |
| 100 nM | 15 | 0.0005 | 10 | 31,176 | 4.401 | 0.9462 | 10,771 |
| 100 nM | 15 | 0.0005 | 10 | 28,857 | 9.23 | 0.9672 | 23,844 |
| 1 μM | 15 | 0.005 | 1 | 37,967 | 18.65 | 0.9108 | 673,498 |
| 1 μM | 15 | 0.005 | 1 | 53,079 | 6.056 | 0.3772 | 301,427 |
| 1 μM | 15 | 0.005 | 1 | 33,540 | 5.694 | 0.0824 | 188,212 |
| 1 μM | 15 | 0.005 | 1 | 28,339 | 8.683 | 0.4756 | 232,590 |
| 1 μM | 15 | 0.005 | 1 | 19,432 | 37.342 | 0.9292 | 707,583 |
| 1 μM | 15 | 0.005 | 1 | 34,840 | 8.236 | 0.3562 | 274,536 |
| 1 μM | 15 | 0.005 | 1 | 31,827 | 10.712 | 0.647 | 320,342 |
| 1 μM | 15 | 0.005 | 1 | 17,634 | 7.62 | 0.247 | 130,013 |
| 1 μM | 15 | 0.005 | 1 | 38,854 | 21.937 | 0.9628 | 814,941 |
| 1 μM | 15 | 0.005 | 1 | 35,320 | 9.275 | 0.2344 | 319,312 |
| 1 μM | 15 | 0.005 | 1 | 31,695 | 6.777 | 0.2378 | 207,258 |
| 1 μM | 15 | 0.005 | 1 | 44,230 | 6.201 | 0.1038 | 269,682 |
| 10 μM | 15 | 0.05 | 0.1 | 26,435 | 5.011 | 0.0642 | 1,307,671 |
| 10 μM | 15 | 0.05 | 0.1 | 55,175 | 4.398 | 0.0792 | 2,382,892 |
| 10 μM | 15 | 0.05 | 0.1 | 26,612 | 4.864 | 0.1028 | 1,267,039 |
| 10 μM | 15 | 0.05 | 0.1 | 40,802 | 5.071 | 0.1428 | 2,010,805 |
| 10 μM | 15 | 0.05 | 0.1 | 25,681 | 5.191 | 0.0318 | 1,324,939 |
| 10 μM | 15 | 0.05 | 0.1 | 57,787 | 7.76 | 0.5746 | 4,152,208 |
| 10 μM | 15 | 0.05 | 0.1 | 34,976 | 5.454 | 0.0782 | 1,880,229 |
| 10 μM | 15 | 0.05 | 0.1 | 64,600 | 6.02 | 0.336 | 3,671,578 |
| 10 μM | 15 | 0.05 | 0.1 | 58,564 | 5.08 | 0.115 | 2,907,960 |
| 10 μM | 15 | 0.05 | 0.1 | 49,243 | 5.909 | 0.260 | 2,781,921 |
| 10 μM | 15 | 0.05 | 0.1 | 37,493 | 6.145 | 0.092 | 2,269,323 |
| 10 μM | 15 | 0.05 | 0.1 | 56,573 | 5.738 | 0.268 | 3,094,765 |
| 10 μM | 15 | 0.05 | 0.1 | 34,060 | 4.782 | 0.236 | 1,548,357 |
| 10 μM | 15 | 0.05 | 0.1 | 50,133 | 5.46 | 0.177 | 2,648,320 |
| 10 μM | 15 | 0.05 | 0.1 | 26,750 | 4.134 | 0.066 | 1,088,123 |
| 10 μM | 15 | 0.05 | 0.1 | 52,148 | 5.042 | 0.164 | 2,543,890 |
| 10 μM | 15 | 0.05 | 0.1 | 47,189 | 5.852 | 0.181 | 2,676,016 |
